# Letter from the editor-in-chief The unavoidable facts of life: changes

**DOI:** 10.1093/ehjdh/ztaf099

**Published:** 2025-08-20

**Authors:** Nico Bruining

**Affiliations:** Department of Cardiology, Erasmus MC, Dr Molewaterplein 40, GD Rotterdam 3015, The Netherlands

Dear readers and digital health enthusiasts,

Time truly flies particularly when things are going very well and certainly in a field which is evolving at light speed. Those of you who have followed us from the beginning have witnessed the remarkable growth of the journal, launched at the end of the ‘COVID year’ of 2020.

I am deeply grateful to the brilliant minds and personalities who supported this endeavour from the very start and helped shape the successes of today. In addition, to the dedicated staff at the European Society of Cardiology, Oxford University Press, and the members and chair of the Digital Health Committee at that time, I want today to especially acknowledge those who were there from the early start in our editorial office in Rotterdam.

Prof. Peter de Jaegere and Paul Cummins played a pivotal role in helping me to develop the initial plans and possible course of the *European Heart Journal—Digital Health*. Soon after, Prof. Joost Lumens joined us. Peter, a leading interventional cardiologist who established the structural heart programme at the Thoraxcenter; Paul, a patient outcomes scientist and founding managing editor of *EuroIntervention*; and Joost, an engineer specializing in computer simulations, formed the ideal team to lay the foundation of the journal.

Looking back, the timing of the launch was perfect. Since then, we have experienced steady growth. Last year, we reached a major milestone: our first impact factor (IF) of 4.0, and recently we received our 2024 IF of 4.4. This accomplishment led to a significant increase in submissions, more than double compared to that of the previous year. As a result, our recent issues have featured a larger number of articles. We are truly grateful for your contributions and extend a heartfelt thank you to all of you, our authors and also thank you reviewers!

**Figure 1:**
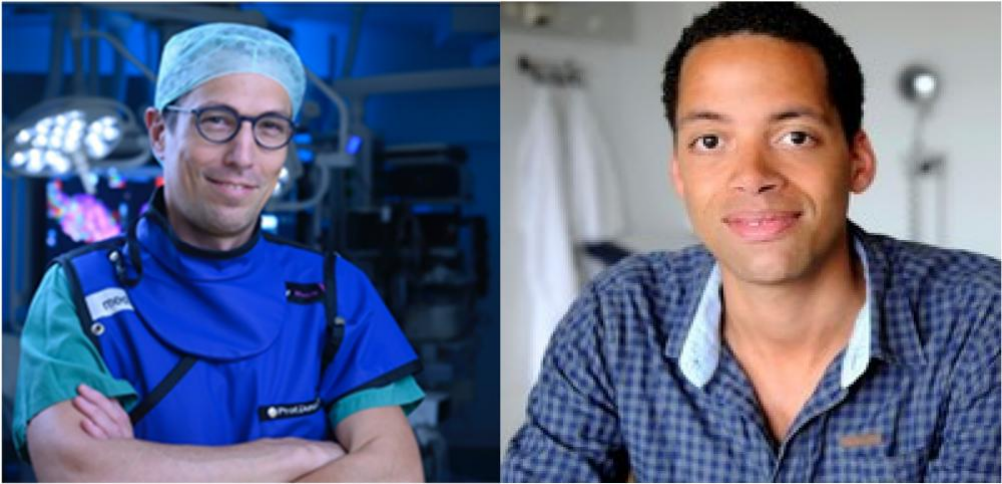
David Duncker and Roderick Scherptong.

To manage the increase in submissions, we are expanding our editorial team. We are very pleased to welcome two new associate/handling editors:

- **Prof. Dr David Duncker**, electrophysiologist at the Hannover Heart Rhythm Center, Hannover Medical School, Germany (*Figure 1*)
- **Dr Roderick Scherptong**, interventional cardiologist at LUMC, Leiden, The Netherlands (*Figure 1*)

Within our Deputy Editors team, we also have Dr Mattie Lenzen, a nurse scientist who oversaw topics within Digital Health closely related to direct patient care, such as remote monitoring, rehabilitation (e.g. apps), and statistical/epidemiological matters. Both Peter and Mattie are highly experienced scientists with long and distinguished careers. Their expertise was instrumental in guiding the journal’s direction. However, as with all things in life, change is inevitable. Both Peter and Mattie have reached their retirement age and have stepped down from their working careers at the Thoraxcenter. As they also expressed their desire to enjoy their next phase of life, which we fully understand, they also retired from the editorial board. It is with great sadness, but with immense gratitude, that we bid them farewell. **Peter and Mattie, thank you for everything!** We are truly indebted to you both.

(*Figure 2*)

**Figure 2:**
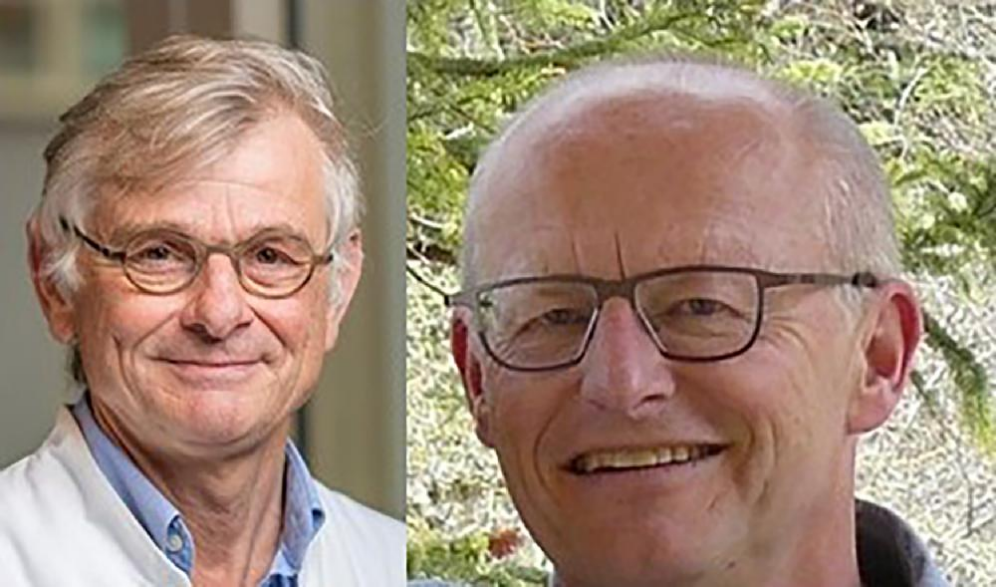
Peter de Jaegere and Mattie Lenzen.

**Figure 3:**
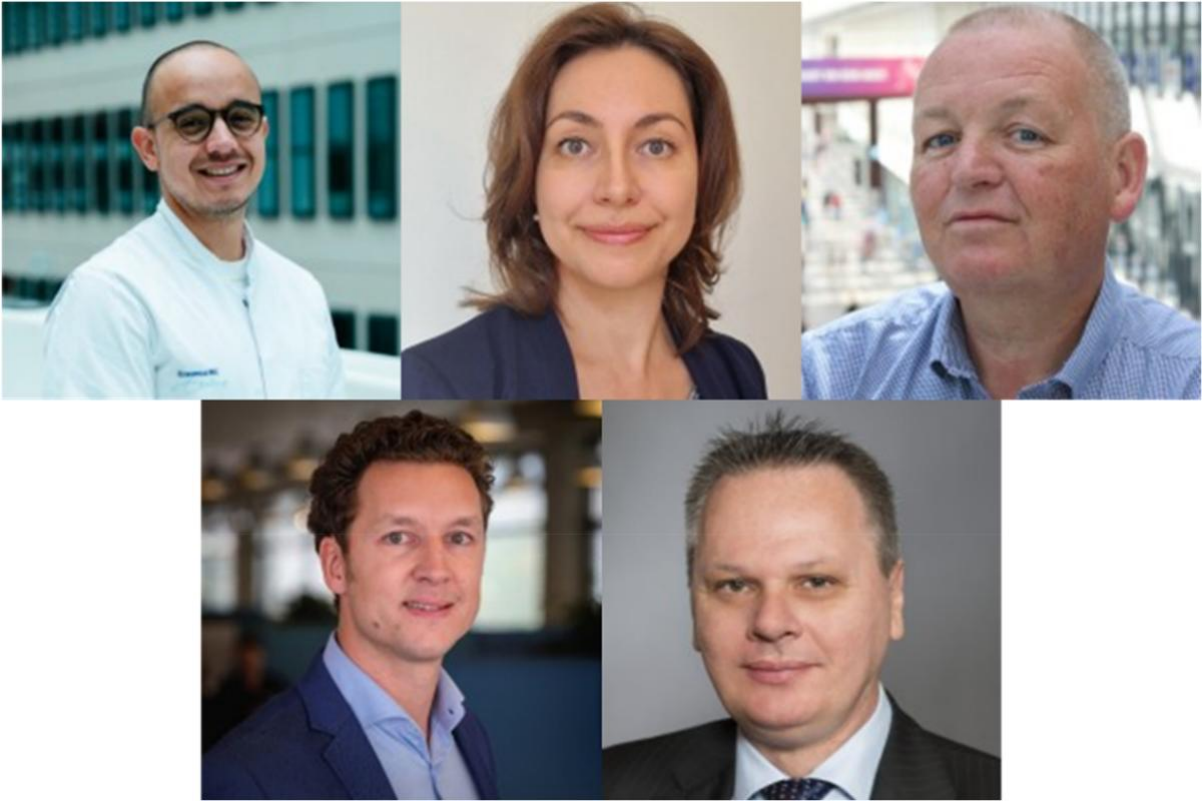
Top: Robert van der Boon, Isabella Kardys, and Paul Cummins. Bottom: Joost Lumens and Nico Bruining.

As a result of these changes, Paul Cummins completed now the Deputy Editors team, which now consists of himself, Robert van der Boon, Isabella Kardys, Joost Lumens, and of course myself (*Figure 3*).

Sincerely yours,

**Nico Bruining**

